# The effect of offering different numbers of colorectal cancer screening test options in a decision aid: a pilot randomized trial

**DOI:** 10.1186/1472-6947-8-4

**Published:** 2008-01-24

**Authors:** Jennifer M Griffith, Carmen L Lewis, Alison RT Brenner, Michael P Pignone

**Affiliations:** 1 Center for Decision Making Research, Cecil Sheps Center for Health Services Research, University of North Carolina-Chapel Hill, Chapel Hill, North Carolina, USA; 2 Division of General Internal Medicine, School of Medicine, University of North Carolina-Chapel Hill, Chapel Hill, North Carolina, USA

## Abstract

**Background:**

Decision aids can improve decision making processes, but the amount and type of information that they should attempt to communicate is controversial. We sought to compare, in a pilot randomized trial, two colorectal cancer (CRC) screening decision aids that differed in the number of screening options presented.

**Methods:**

Adults ages 48–75 not currently up to date with screening were recruited from the community and randomized to view one of two versions of our previously tested CRC screening decision aid. The first version included five screening options: fecal occult blood test (FOBT), sigmoidoscopy, a combination of FOBT and sigmoidoscopy, colonoscopy, and barium enema. The second discussed only the two most frequently selected screening options, FOBT and colonoscopy. Main outcomes were differences in screening interest and test preferences between groups after decision aid viewing. Patient test preference was elicited first without any associated out-of-pocket costs (OPC), and then with the following costs: FOBT-$10, sigmoidoscopy-$50, barium enema-$50, and colonoscopy-$200.

**Results:**

62 adults participated: 25 viewed the 5-option decision aid, and 37 viewed the 2-option version. Mean age was 54 (range 48–72), 58% were women, 71% were White, 24% African-American; 58% had completed at least a 4-year college degree. Comparing participants that viewed the 5-option version with participants who viewed the 2-option version, there were no differences in screening interest after viewing (1.8 vs. 1.9, t-test *p *= 0.76). Those viewing the 2-option version were somewhat more likely to choose colonoscopy than those viewing the 5-option version when no out of pocket costs were assumed (68% vs. 46%, *p *= 0.11), but not when such costs were imposed (41% vs. 42%, *p *= 1.00).

**Conclusion:**

The number of screening options available does not appear to have a large effect on interest in colorectal cancer screening. The effect of offering differing numbers of options may affect test choice when out-of-pocket costs are not considered.

## Background

Colon cancer screening is effective in reducing the incidence and mortality from colorectal cancer (CRC), but is currently underutilized, with national self-reported screening rates of 57.3% [1]. Several methods of screening are available and recommended by guideline-issuing organizations, including fecal occult blood tests, sigmoidoscopy, colonoscopy, and radiological screening with barium enema or CT colonography [2]. These tests differ in several respects, including preparation required, frequency of screening, amount of discomfort and time required, chance of complications, out-of-pocket costs, and efficacy in preventing CRC or death from CRC [3].

Sub-optimal screening rates have led to many efforts to develop interventions to increase screening. Patient decision aids have been shown to increase screening rates and improve decision making for CRC screening [3,4]. Decision aids help patients become aware of colon cancer as a salient health issue; provide information about testing options, including their benefits and downsides; and help patients to discuss screening with their providers.

One important issue when developing decision aids is the decision about how much total information to include. The amount of information that is presented should reflect moral, ethical, and legal obligations to provide a balanced and informative tool. Practically, developers have to balance the benefits of presenting more potentially useful information against the downsides of creating a tool that is too long and thus impractical or the potential that providing more information could lead to poorer decision making processes, as demonstrated by Schwartz [5] and Iyengar [6]. Two specific challenges in CRC screening are the questions of how many different testing strategies should be included in the decision aid, and whether the decision aid should include information about out-of-pocket costs. In terms of the number of tests, some experts argue that all plausible testing options should be included [7,8]. Conversely, the argument for presenting fewer testing options is supported by research that suggests increasing number of choices can result in increased difficulty with decision making and poor decision outcomes [5,6]. With respect to the issue of costs, two views could be taken: those who favor inclusion of cost information could argue that such information is crucial to decision making because it is important to patients and should be used to compare the value of screening versus other important uses; those opposing inclusion could argue that it is practically difficult to provide information about costs that is salient on the individual level because of the differences in insurance coverage in the US.

To help address these issues, we sought to compare, in a pilot randomized trial, the effect of providing information about different numbers of CRC screening test options on interest in screening and screening test preferences, with and without information about out-of-pocket costs.

## Methods

### Participant Eligibility

Adults ages 48–75 not currently up-to-date with CRC screening (FOBT within the last year, sigmoidoscopy or barium enema in the last 5 years, colonoscopy within the last 10 years) were recruited to the UNC Decision Support Lab (DSL) from our DSL participant registry and through mass media recruiting (newspapers and email listservs). Individuals with a personal or family history of CRC, polyps, or inflammatory bowel disease were excluded from the study. This study was reviewed and approved by the Institutional Review Board at UNC-Chapel Hill.

Eligible patients completed informed consent at the beginning of their study session. They were randomized to view one of two decision aids in the lab: a 5-option version or a 2-option version. We used the random-number generator in STATA to create sequential study assignment envelopes. The envelopes were opened by the research assistant at the beginning of each participant session and determined which version of the decision aid participants viewed.

### Decision Aid

The development of the full, 5-option version of the decision aid has been described previously [3]. The five screening options included are fecal occult blood test (FOBT), sigmoidoscopy, a combination of FOBT and sigmoidoscopy, colonoscopy, and barium enema. The decision aid contained introductory information about colon cancer and the screening decision, more detailed information about each of the tests, and comparative information for those who wished to decide between different tests. Viewing the entire decision aid, which was on DVD, required approximately 30 minutes. Participants were required to view the introductory portion of the decision aid, which lasted approximately 7 minutes, and were able to view additional information on each test by navigating the DVD's chapter menu.

The second, 2-option decision aid was a shortened (approximately 15 minutes total content) version of the full decision aid that included only the two options, FOBT and colonoscopy, most frequently chosen by patients in our previous study [3,4]. The required introductory segment for this version was approximately 5 minutes. Participants could then view additional information on FOBT and colonoscopy.

The section comparing the different tests in terms of how often tests need to be completed, preparation for tests, time required for tests, ability of test to find polyps and cancer, discomfort during tests, and chance of complications was included in both versions. Both versions ended in a stage-based assessment of screening readiness [4].

### Measures

The full questionnaires are included as Additional file 1. Our main outcomes were differences in screening interest and patient test preferences between versions of the decision aid. As a pilot study, our sample size was based on resources available, not a formal power calculation. This study was conducted as a lab-based study outside of a clinical setting and participants were told to respond to the best of their ability when indicating a test preference. We did not measure actual test completion, so test decisions should be interpreted as hypothetical decisions.

Participants completed questionnaires before and after viewing the decision aid. For screening interest, we used a single item with a 5-point Likert response scale that was assessed pre and post decision aid viewing.

Participant test preferences were also elicited after viewing the decision aid, first without any associated out-of-pocket costs, and then with the following costs: FOBT-$10, sigmoidoscopy-$50, barium enema-$50 and colonoscopy-$200. These out-of-pocket cost levels were based, partially, on estimates of co-payments required in the Medicare program [9].

We also measured several other secondary outcomes, including knowledge, decision satisfaction, and decisional conflict. We assessed knowledge using 3 questions that were administered pre and post decision aid viewing: 1 point was awarded for a correct response, resulting in scores ranging from 0 – 3. Decision Satisfaction was measured with a 6 item scale by Wills and Holmes-Rovner [10] after viewing the decision aid; scores ranged from 6 – 30; higher scores corresponded to higher satisfaction. O'Connor's 16-item Decisional Conflict Scale [11] was also assessed after viewing the decision aid. Scores calculated by adding the total for the responses and dividing by 16, scores ranged from 1 – 5; lower scores were associated with lower conflict.

We also assessed several subjective measures of decision aid content after decision aid viewing, including the amount of information presented, balance, and overall impression. These measures also employed Likert response scales and were drawn from previous studies by our group [12]. Higher scores were associated with better subjective impressions of amount of information on advantages and disadvantages of screening, preparation for discussion with the doctor, and preparation for making a decision. For the subjective balance of the decision aid we asked: Do you think the video was: strongly in favor of screening, somewhat in favor of screening, neither in favor of nor against screening, somewhat against screening, strongly against screening.

### Analysis

We used means and proportions to report our descriptive statistics. To compare the different versions of the decision aid, we used t-tests and Wilcoxon rank sum for continuous measures and chi-square and Fisher's exact tests for proportions. When the results of the non-parametric tests did not differ from the parametric ones, we reported the parametric statistics. Statistical tests were considered significant with a p-value of 0.05 or less, but results that did not reach statistically significant were not necessarily considered clinically unimportant because of our small sample size. Because our small sample size did not permit equal distribution of potential confounders, we also performed multivariate analyses, using linear regression and logistic regression, to account for baseline differences in the intervention and control groups.

The study was approved by the University of North Carolina Biomedical Institutional Review Board.

## Results

**Figure 1:**
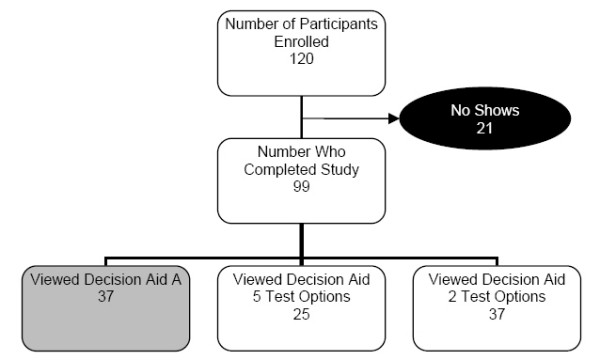
Study Randomization.

We contacted 175 participants from our database, 120 enrolled and 99 completed the study: 25 viewed the 5-option version, and 37 viewed the 2-option version; 37 others were enrolled in a different arm of the trial that compared a different decision aid and are not reported further here (Figure 1). Among the 62 participants, mean age was 54 (range 48–72), 58% were women, 71% were White, 24% African-American, 58% had completed at least a 4-year college degree. Most (58%) had never discussed CRC screening with their doctor. Most characteristics were relatively well-balanced between groups, except that a greater proportion of women viewed the 5-option version than the 2-option version. (Table 1)

**Table 1:** Demographic Information

	Overall (n = 62)	5-option version (n = 25)	2-option version (n = 37)
Mean age, years (range)	54	55	54
% female	58	68	51
% White	71	68	73
% African-American	24	28	22
% reporting 4 or more years of college	58	60	57
% reporting not having discussed screening with their doctor	58	52	62
Baseline Knowledge Score*	1.6	1.7	1.5
Baseline Screening Interest**	1.9	1.8	1.9

* Mean baseline knowledge scores; range 0–3

** Mean baseline screening interest; range 1–5 with 1 indicated definitely interested in being screened; 2 indicated probably interested in being screened

### Effect of number of screening options on screening interest and test preferences between groups

When comparing participants that viewed the 5-option version with participants who viewed the 2-option version, there were no differences in interest in screening after viewing the decision aid (1.8 vs. 1.9, t-test *p *= 0.76).

Patient preferences for type of screening test, according to decision aid version viewed, are presented in Table 2. The proportion of patients choosing colonoscopy over other test options differed somewhat between those viewing the 5-option version and 2-option version when no out-of-pocket costs were assumed, although the results did not reach statistical significance (68% vs. 46%, χ^2 ^*p *= 0.11). These differences were not observed when out-of-pocket costs were imposed (41% vs. 42%, χ^2 ^*p *= 1.00).

**Table 2:** Preferred test choice without and with out-of-pocket costs

**Test Type**	**5-Option Version **(n = 25)		**2-Option Version **(n = 37)	
	**Without OPC**	With OPC*	**Without OPC**	With OPC**
FOBT	21%	17%	32%	54%
Sigmoidoscopy	4%	8%	-	-
FOBT & Sigmoidoscopy	21%	21%	-	-
Colonoscopy	46%	42%	68%	41%
Barium Enema	4%	8%	-	-
No Screening	4%	4%	-	5%

* Proportion of participants choosing colonoscopy with and without out-of-pocket costs for those viewing the 5-option version (p = 0.31 McNemar's test)

** Proportion of participants choosing colonoscopy based on out-of-pocket costs for the 2-option version (p = 0.0016)

^† ^Proportion participants selecting colonoscopy between groups without out-of-pocket costs (χ^2 ^*p *= 0.11)

^†† ^Proportion participants selecting colonoscopy between groups with out-of-pocket costs (χ^2 ^*p *= 1.00)

### Effect of out-of-pocket costs within groups

The proportion choosing colonoscopy appeared to be affected by out-of-pocket costs for those viewing the 2-option version, but not for those viewing the 5-option version. For those who viewed the 2-option version, 68% of participants selected colonoscopy without out-of-pocket costs, but only 41% selected colonoscopy when out-of-pocket costs were added. For the 5-option version the proportions were 46% and 42% respectively. (Table 2)

### Secondary outcomes between groups

Post-video knowledge scores also did not differ significantly (2.1 vs. 2.0, t-test *p *= 0.75; 36% vs. 32% responding correctly to all questions) between the two groups. Decisional conflict (mean 1.9 in each group, *p *= 0.43) and decision satisfaction (mean 25 in each group, *p *= 0.78) were also similar between groups. (Table 3)

**Table 3:** Secondary Outcomes: Knowledge, Decisional Conflict, Decisional Satisfaction and Subjective Measures

	5-option version (n = 25)	2-option version (n = 37)	*p*-value
Knowledge	2.1	2.0	0.75
Decisional Conflict	1.9	1.9	0.43
Decisional Satisfaction	25.0	25.0	0.78
Ability to help participate in deciding about screening	4.2	4.0	0.33
Length of Video	3.0	3.0	0.35
Amount of Information on Benefits	2.9	2.8	0.41
Amount of Information on Disadvantages	2.5	2.6	0.49
Ability to help prepare to talk with doctor	4.2	3.9	0.11
Ability to help prepare to make a decision	4.5	3.9	0.03

For the subjective measures of decision aid content there were no significant differences between the groups in regards to the amount of information on advantages (2.9 vs. 2.8, t-test *p *= 0.41) or disadvantages (2.5 vs. 2.6, t-test *p *= 0.49), or in the ability of the decision aid to help prepare people to talk with their doctors (4.2 vs. 3.9, t-test *p *= 0.11). There was a significant difference between groups in the subjective measure of the decision aid's ability to help people prepare to make a decision with the 5-option version being rated higher than the 2-option version (4.5 vs. 3.9, t-test *p *= 0.03).

There was no difference between the groups in the rating of the videos balance (Fisher's exact *p *= 0.32). Most rated the video strongly in favor of screening (5-option version 84% vs. 2-option version 73%) or somewhat in favor of screening (5-option version 12% vs. 2-option version 19%). Three participants (8%) in the 2-option version rated the decision aid as neither in favor of nor against screening.

### Multivariate analyses

Because there were differences between the groups at baseline, we performed multivariate analyses to examine the effect of individual co-variates (age, sex, educational level, previous screening discussion) on the relationship between decision aid version and our outcomes. After controlling for age, sex, education, and previous screening discussion, we did not find that any of these variables affected the (lack of) relationship between decision aid version and our outcomes seen in bi-variate analyses.

## Discussion

The number of screening options presented in a decision aid does not appear have a large effect on interest in colorectal cancer screening. Test choice appeared to differ modestly (although the difference did not reach statistical significance) between the 5-option and 2-option version when no out-of-pocket costs were assumed. This difference was not apparent when participants were asked to assume modest out-of-pocket costs.

Those participants viewing the two-test option decision aid were somewhat more sensitive to out-of-pocket costs than those viewing the 5-option version, perhaps because the two tests included, FOBT and colonoscopy, were those that differed most in terms of potential out-of-pocket costs. By focusing the decision on these two options, viewers may have been drawn to weigh the out-of-pocket costs more heavily than if they had been exposed to a wider range of options.

The psychology literature has examined the relationship between the number of choices offered and its effect on decision making. Schwartz' [5] reviewed much of this work and concluded that providing more choices could lead to poorer decision making processes in both health-related and non-health-related contexts. Work in consumer psychology by Iyengar [6] and colleagues demonstrated that the provision of extensive choices led to dissatisfaction and decision regret which also supports this premise. Using survey data, Lafata and colleagues found that patients who reported being offered a choice of CRC screening modality were less likely to have complete a CRC screening test in the last 5 years [13].

Based on this work, some cancer screening researchers and policymakers have questioned the effect of offering patients more than one option for how to be screened, suggesting that overall screening rates might be higher if the patient is only presented with one method. Although our study did not directly test this question, we did not see major differences in screening interest between offering two or five options. Randomized trials from Italy and Australia have compared the effect of offering one test versus choice of FOBT or sigmoidoscopy and did not find important differences in screening rates [14,15].

Our findings, with respect to test preferences and the effect of out-of-pocket costs, are consistent with some of our previous work as well as work of others [16]. We had previously shown that out-of-pocket costs affected patient preferences for CRC screening when considering three options: FOBT alone, sigmoidoscopy alone, or the combination of FOBT and sigmoidoscopy [17]. Leard and colleagues reported that they did not find cost to be an important factor in CRC screening decision making, but they did not compare the effect of providing or not providing such information [16,18]. Like Leard and colleagues, we found that nearly all participants expressed a preference for some form of screening.

Our study has several limitations. First, it is a small randomized trial. We did not have sufficient power to confirm or exclude modest, but potentially meaningful, differences in effect. We also did not have a large enough sample size to ensure equal distribution of potential confounders, requiring adjustment in multivariate analysis. However, adjusting for these baseline differences did not change the relationships noted in the bivariate analyses. In addition, we did not assess the amount of information that participants viewed beyond the initial required introduction section of each program. Participants were reporting their hypothetical interests and preferences for screening after exposure to a decision aid. The relationship between interest and preferences and actual test ordering or completion is imperfect. Future studies should examine the effect of the number of test options on actual test completion, including whether the "preferred" test is also the one ordered and completed.

The tests discussed in the decision aid did not include such newer screening options as fecal immunochemical tests, stool DNA, or CT colonography, all of which may have produced different preference patterns. Finally, we did not randomize the order of questions about preferences based on inclusion or exclusion of out-of-pocket costs. It is possible that we would have obtained somewhat different results if we had elicited preferences with such costs before eliciting them without such costs and other co-variates.

## Conclusion

Based on our results, we conclude that the number of test options has no major effect on interest in CRC screening. Future research should consider assessing patient preferences and assessing individual level change when given two sets of options (all possible tests or only FOBT and colonoscopy) to determine whether patients are more or less likely to get screened. Out-of-pocket costs can have important effects on test preferences, at least when choices are constrained to the options of FOBT and colonoscopy. Insurers and payers should consider the effects of out-of-pocket costs when developing their screening policies, particularly if they wish to encourage screening [19-23].

## List of Abbreviations

CRC – colorectal cancer

FOBT – Fecal Occult Blood Test

## Competing interests

The author(s) declare that they have no competing interests.

## Authors' contributions

JMG participated in the design, management of data collection, and initial data analysis for the study and drafted and revised the manuscript. MPP conceived of the study, and participated in its design, statistical analyses, interpretation of results and drafting and revising the manuscript. CLL helped interpret results and draft and revise the manuscript. ATB participated in study design and data collection. All authors read and approved the final manuscript.

## Pre-publication history

The pre-publication history for this paper can be accessed here:



## Supplementary Material

Additional file 1 CHOICE2 decision aid study surveys. The pre and post decision aid surveys used in CHOICE2 study. Click here for file

## References

[B1] (2006). Increased use of colorectal cancer tests – United States, 2002 and 2004. Mmwr.

[B2] (2002). Screening for colorectal cancer: recommendation and rationale. Annals of internal medicine.

[B3] Kim J, Whitney A, Hayter S, Lewis C, Campbell M, Sutherland L, Fowler B, Googe S, McCoy R, Pignone M (2005). Development and initial testing of a computer-based patient decision aid to promote colorectal cancer screening for primary care practice. BMC medical informatics and decision making.

[B4] Pignone M, Harris R, Kinsinger L (2000). Videotape-based decision aid for colon cancer screening. A randomized, controlled trial. Annals of internal medicine.

[B5] Schwartz B (2004). The Paradox of Choice: why more is less. HarperCollins.

[B6] Iyengar SS, Lepper MR (2000). When choice is demotivating: can one desire too much of a good thing?. Journal of personality and social psychology.

[B7] Jepson RG, Forbes CA, Sowden AJ, Lewis RA (2001). Increasing informed uptake and non-uptake of screening: evidence from a systematic review. Health Expect.

[B8] Raffle AE (2001). Information about screening – is it to achieve high uptake or to ensure informed choice?. Health Expect.

[B9] Does Medicare cover colorectal cancer screenings?. http://www.medicareinteractive.org.

[B10] Wills CE, Holmes-Rovner M (2003). Preliminary validation of the Satisfaction With Decision scale with depressed primary care patients. Health Expect.

[B11] O'Connor AM (1995). Validation of a decisional conflict scale. Med Decis Making.

[B12] Griffith JM, Mitcher M, Folwer FJ, Lewis C, Pignone M Should a Colon Cancer Screening Decision Aid Include the Option of No Testing? A Controlled Trial Comparing Two Videos. BMC Journal Medical Informatics and Decision Making.

[B13] Lafata JE, Divine G, Moon C, Williams LK (2006). Patient-Physician Colorectal Cancer Screening Discussions and Screening Use. American journal of preventive medicine.

[B14] (2006). A comparison of colorectal neoplasia screening tests a multicentre community-based study of the impact of consumer choice. The Medical journal of Australia.

[B15] Segnan N, Senore C, Andreoni B, Arrigoni A, Bisanti L, Cardelli A, Castiglione G, Crosta C, DiPlacido R, Ferrari A (2005). Randomized trial of different screening strategies for colorectal cancer: patient response and detection rates. Journal of the National Cancer Institute.

[B16] Pignone M (2007). Patient preferences for colon cancer screening: the role of out-of-pocket costs. The American journal of managed care.

[B17] Pignone M, Bucholtz D, Harris R (1999). Patient preferences for colon cancer screening. J Gen Intern Med.

[B18] Leard LE, Savides TJ, Ganiats TG (1997). Patient preferences for colorectal cancer screening. The Journal of family practice.

[B19] Braithwaite RS, Rosen AB (2007). Linking cost sharing to value: an unrivaled yet unrealized public health opportunity. Annals of internal medicine.

[B20] Goldman DP, Joyce GF, Escarce JJ, Pace JE, Solomon MD, Laouri M, Landsman PB, Teutsch SM (2004). Pharmacy benefits and the use of drugs by the chronically ill. Jama.

[B21] Motheral B, Fairman KA (2001). Effect of a three-tier prescription copay on pharmaceutical and other medical utilization. Medical care.

[B22] Tamblyn R, Laprise R, Hanley JA, Abrahamowicz M, Scott S, Mayo N, Hurley J, Grad R, Latimer E, Perreault R (2001). Adverse events associated with prescription drug cost-sharing among poor and elderly persons. Jama.

[B23] Thomas CP (2003). Incentive-based formularies. The New England journal of medicine.

