# Supplementary material for: The effect of offering different numbers of colorectal cancer screening test options in a decision aid: a pilot randomized trial
Source: BMC Med Inform Decis Mak. 2008 Jan 24;8:4. doi: 10.1186/1472-6947-8-4 (PMC2259331; doi:10.1186/1472-6947-8-4)
Supplement: Additional file 1 — CHOICE2 decision aid study surveys. The pre and post decision aid surveys used in CHOICE2 study. [file 1472-6947-8-4-S1.doc]

| **SHARED DECISION PROGRAM**  **Colon Cancer Screening**  **Pre-Video Survey** | | | | | |
| --- | --- | --- | --- | --- | --- |
|  | |  |  |  |  |
| ***For each of the following questions please select one response. There are no right or wrong answers.*** | | | | | |
|  | |  |  |  |  |
| 1. What is your date of birth? ____/_____/_______   ^MM DD YYYY^ | | | | | |
|  | | | | | |
| 1. Are you: | | | | | |
|  | Male | |  |  |  |
|  | Female | | |  |  |
|  | | | | | |
| 1. What is the highest grade or level of school that you have completed?  (check one) | | | | | |
|  | 8th grade or less | |  |  |  |
|  | Some high school, but did not graduate | | |  |  |
|  | High school graduate or GED | |  |  |  |
|  | Some college or 2-year degree | | |  |  |
|  | 4-year college graduate | | |  |  |
|  | More than 4-year college degree | | |  |  |
|  | | | | | |
| 1. How would you describe yourself? (check the one that best describes you) | | | | | |
|  | American Indian or Alaskan Native | | |  |  |
|  | Asian or Pacific Islander | | |  |  |
|  | Black or African-American | | |  |  |
|  | Latino | | |  |  |
|  | White | | |  |  |
|  | Another race or multiracial (write in) _____________________________ | | | | |
|  | | | | | |
| 1. Have you ever discussed colon cancer screening with your doctor? | | | | | |
|  | Yes | |  |  |  |
|  | No | |  |  |  |
|  | |  |  |  |  |
| 1. Are you interested in being screened for colon cancer in the next 6 months? (check one)? | | | | | |
|  | Definitely interested in being screened | | | |  |
|  | Probably interested in being screened | | | |  |
|  | Not sure if I am interested or not | | |  |  |
|  | Probably not interested in being screened | | | |  |
|  | Definitely not interested in being screened | | | |  |
|  | | | | | |
| 1. Do you intend to ask your doctor about being screened for colon cancer in the next 6 months? (check one) | | | | | |
|  | Definitely intend to ask for screening | | | |  |
|  | Probably intend to ask for screening | | | |  |
|  | Probably don’t intend to ask for screening | | | |  |
|  | Definitely don’t intend to ask for screening | | | |  |
|  | |  |  |  |  |

| 1. At what age do you think people at average risk who want to be screened should start getting regular screening tests for colon cancer? (check one) | | | | | |
| --- | --- | --- | --- | --- | --- |
|  | 35 | |  |  |  |
|  | 40 | | |  |  |
|  | 45 | | |  |  |
|  | 50 | | |  |  |
|  | 55 | |  |  |  |
|  | |  |  |  |  |
| 1. About how many polyps will develop into cancer if not removed? (check one) | | | | | |
|  | Most will | |  |  |  |
|  | About half | | |  |  |
|  | Most will not | |  |  |  |
|  | |  |  |  |  |
| 1. Out of 100 adults, about how many will develop colon cancer in their lifetime? (check one) | | | | | |
|  | Fewer than 10 | |  |  |  |
|  | 10-19 | | |  |  |
|  | 20-29 | |  |  |  |
|  | 30 or more | |  |  |  |
|  | |  |  |  |  |

| **SHARED DECISION PROGRAM**  **Colon Cancer Screening**  **Post-Video Survey** | | | | | |
| --- | --- | --- | --- | --- | --- |
|  | |  |  |  |  |
| ***For each of the following questions, please mark the answer that you feel best described your thoughts and feelings about the video you just viewed. There are no right or wrong answers.***  ***Note that questions shown in italics were only given to participants who viewed the 5 option version of the Decision Aid.*** | | | | | |
|  | |  |  |  |  |
| 1. The makers of this video want to show why patients, and not just doctors, should participate in deciding about colon cancer testing. How would you rate the video’s ability to help you to participate in deciding about screening? (check one) | | | | | |
|  | Poor | |  |  |  |
|  | Fair | |  |  |  |
|  | Good | |  |  |  |
|  | Very Good | |  |  |  |
|  | Excellent | |  |  |  |
|  | |  |  |  |  |
| 1. How would you rate the **length** of the video? (check one) | | | | | |
|  | Should be much shorter | |  |  |  |
|  | Should be a little shorter | |  |  |  |
|  | About right | |  |  |  |
|  | Could be a little longer | |  |  |  |
|  | Could be much longer | |  |  |  |
|  | |  |  |  |  |
| 1. The **amount** of information about the possible **benefits** of colon cancer screening was: (check one) | | | | | |
|  | Much less than I wanted | |  |  |  |
|  | A little less than I wanted | |  |  |  |
|  | About right | |  |  |  |
|  | A little more than I wanted | |  |  |  |
|  | Much more than I wanted | |  |  |  |
|  | |  |  |  |  |
| 1. The **amount** of information about the possible **disadvantages** of colon cancer screening was: (check one) | | | | | |
|  | Much less than I wanted | |  |  |  |
|  | A little less than I wanted | |  |  |  |
|  | About right | |  |  |  |
|  | A little more than I wanted | |  |  |  |
|  | Much more than I wanted | |  |  |  |
|  | |  |  |  |  |
| 1. A goal of this video is to help people **prepare to talk with their doctors** about colon cancer screening. How would you rate the video’s ability to help people prepare to talk with their doctors? (check one) | | | | | |
|  | Poor | |  |  |  |
|  | Fair | |  |  |  |
|  | Good | |  |  |  |
|  | Very Good | |  |  |  |
|  | Excellent | |  |  |  |
|  | |  |  |  |  |

| 1. Another goal of this video is to help people **prepare to make a decision** about colon cancer screening. How would you rate the video’s ability to prepare people to make a decision? (check one) | | | | | |  |
| --- | --- | --- | --- | --- | --- | --- |
|  | Poor | |  |  |  |  |
|  | Fair | |  |  |  |  |
|  | Good | |  |  |  |  |
|  | Very Good | |  |  |  |  |
|  | Excellent | |  |  |  |  |
|  | |  |  |  |  |  |
| 1. Do you think the video was: (check one) | | | | | |  |
|  | Strongly in favor of screening | | |  |  |  |
|  | Somewhat in favor of screening | | |  |  |  |
|  | Neither in favor nor against screening | | |  |  |  |
|  | Somewhat against screening | | |  |  |  |
|  | Strongly against screening | | |  |  |  |
|  | |  |  |  |  |  |
| 1. In terms of the different ways of being screened, do you think the video favored FOBT (Fecal Occult Blood Test)? | | | | | |  |
|  | Yes | | |  |  |  |
|  | No | | |  |  |  |
|  | | | | | |  |
| 1. *In terms of the different ways of being screened, do you think the video favored sigmoidoscopy?* | | | | | |  |
|  | *Yes* | | |  |  |  |
|  | *No* | | |  |  |  |
|  |  | | |  |  |  |
| 1. In terms of the different ways of being screened, do you think the video favored colonoscopy? | | | | | |  |
|  | Yes | | |  |  |  |
|  | No | | |  |  |  |
|  |  | | |  |  |  |
| 1. *In terms of the different ways of being screened, do you think the video favored radiological screening (barium enema)?* | | | | | |  |
|  | *Yes* | | |  |  |  |
|  | *No* | | |  |  |  |
|  |  | | |  |  |  |
| 1. In terms of the different ways of being screened, do you think the video did not favor any approach over the others? | | | | | |  |
|  | Yes | | |  |  |  |
|  | No | | |  |  |  |
|  |  | | |  |  |  |
| ***For each of the following questions, please mark the response you feel best answers the question.*** | | | | | |  |
|  | |  |  |  |  |  |
| 1. At what age should people at average risk who want to be screened start getting regular screening tests for colon cancer? (check one) | | | | | |  |
|  | 35 | | |  |  |  |
|  | 40 | | |  |  |  |
|  | 45 | | |  |  |  |
|  | 50 | | | |  |  |
|  | 55 | | | |  |  |
|  | |  |  |  |  |  |
| 1. About how many polyps will develop into cancer if not removed? (check one) | | | | | |  |
|  | Most will | | | | |  |
|  | About half will | | |  |  |  |
|  | Most will not | | |  |  |  |
|  | |  |  |  |  |  |
| 1. Out of 100 adults, about how many will develop colon cancer in their lifetime? (check one) | | | | | |  |
|  | Fewer than 10 | | | | |  |
|  | 10-19 | | |  |  |  |
|  | 20-29 | | |  |  |  |
|  | 30 or more | | | |  |  |
|  | |  |  |  |  |  |
| ***For the following questions please indicate your decision and choices regarding colon cancer screening.*** | | | | | |  |
|  | |  |  |  |  |  |
| 1. Are you interested in being screened for colon cancer in the next 6 months? (check one) | | | | | |  |
|  | Definitely interested in being screened | | | | |  |
|  | Probably interested in being screened | | | | |  |
|  | Not sure if I am interested or not | | | | |  |
|  | Probably not interested in being screened | | | | |  |
|  | Definitely not interested in being screened | | | | |  |
|  | |  |  |  |  |  |
| 1. Do you intend to ask your doctor about being screened for colon cancer in the next 6 months? (check one) | | | | | |  |
|  | Definitely intend to ask for screening | | | | |  |
|  | Probably intend to ask for screening | | | | |  |
|  | Not sure if I intend to ask for screening | | | | |  |
|  | Probably don't intend to ask for screening | | | | |  |
|  | Definitely don't intend to ask for screening | | | | |  |
|  | |  |  |  |  |  |
| 1. If you were going to be tested, which test would you want to have assuming **THERE WERE NO** out-of-pocket costs? (check one) | | | | | |  |
|  | FOBT (Fecal Occult Blood Test) each year alone | | | | |  |
|  | Sigmoidoscopy every 5 years alone | | |  |  |  |
|  | FOBT each year and sigmoidoscopy every 5 years | | | |  |  |
|  | Colonoscopy every 10 years | | | |  |  |
|  | Barium enema every 5 years | | |  |  |  |
|  | Do not intend to be tested | | |  |  |  |
|  | |  |  |  |  |  |
| 1. If you were going to be tested, which test would you want to have assuming **THERE WERE** the following out-of-pocket costs? (check one) | | | | | |  |
|  | FOBT (Fecal Occult Blood Test) each year alone, cost $10 | | | | |  |
|  | Sigmoidoscopy every 5 years alone, $50 | | |  |  |  |
|  | FOBT each year and sigmoidoscopy every 5 years, cost $10 each year, $50 every 5^th^ year | | | | | |
|  | Colonoscopy every 10 years, cost $200 | | | |  |  |
|  | Barium enema every 5 years, cost, $50 | | |  |  |  |
|  | Do not intend to be tested | | |  |  |  |
|  | |  |  |  |  |  |

| ***Please answer the following questions about your colon cancer screening decision. Please indicate to what extent each statement is true for you AT THIS TIME. There are no right or wrong answers.*** | | | | |
| --- | --- | --- | --- | --- |
|  |  |  |  |  |
| 1. I was adequately informed about the different types of colon cancer screening tests available. | | | | |
|  |  |  |  |  |
| Strongly disagree | Disagree | Neither agree nor disagree | Agree | Strongly agree |
|  |  |  |  |  |
| 1. The decision I made about colon cancer screening was the best decision possible for me personally. | | | | |
|  |  |  |  |  |
| Strongly disagree | Disagree | Neither agree nor disagree | Agree | Strongly agree |
|  |  |  |  |  |
| 1. My decision colon cancer screening was consistent with my personal values. | | | | |
|  |  |  |  |  |
| Strongly disagree | Disagree | Neither agree nor disagree | Agree | Strongly agree |
|  |  |  |  |  |
| 1. I expect to successfully carry out the decision I made about colon cancer screening. | | | | |
|  |  |  |  |  |
| Strongly disagree | Disagree | Neither agree nor disagree | Agree | Strongly agree |
|  |  |  |  |  |
| 1. I had as much input as I wanted on the choice of a colon cancer screening test. | | | | |
|  |  |  |  |  |
| Strongly disagree | Disagree | Neither agree nor disagree | Agree | Strongly agree |
| 1. I am satisfied with the decision that was made about screening for colon cancer. | | | | |
|  |  |  |  |  |
| Strongly disagree | Disagree | Neither agree nor disagree | Agree | Strongly agree |
|  |  |  |  |  |
| 1. This decision about colon cancer screening is easy for me to make. | | | | |
|  |  |  |  |  |
| Strongly disagree | Disagree | Neither agree nor disagree | Agree | Strongly agree |
|  |  |  |  |  |
| 1. I'm sure what to do in this decision about colon cancer screening. | | | | |
|  |  |  |  |  |
| Strongly disagree | Disagree | Neither agree nor disagree | Agree | Strongly agree |
|  |  |  |  |  |
| 1. It's clear what choice about colon cancer screening is best for me. | | | | |
|  |  |  |  |  |
| Strongly disagree | Disagree | Neither agree nor disagree | Agree | Strongly agree |
|  |  |  |  |  |

| 1. I'm aware of the options I have in this decision about colon cancer screening. | | | | |
| --- | --- | --- | --- | --- |
|  |  |  |  |  |
| Strongly disagree | Disagree | Neither agree nor disagree | Agree | Strongly agree |
|  |  |  |  |  |
| 1. I feel I know the advantages of each option for colon cancer screening. | | | | |
|  |  |  |  |  |
| Strongly disagree | Disagree | Neither agree nor disagree | Agree | Strongly agree |
|  |  |  |  |  |
| 1. I feel I know the disadvantages of each option for colon cancer screening. | | | | |
|  |  |  |  |  |
| Strongly disagree | Disagree | Neither agree nor disagree | Agree | Strongly agree |
|  |  |  |  |  |
| 1. I am clear about how important the advantages are to me in the decision about colon cancer screening. | | | | |
|  |  |  |  |  |
| Strongly disagree | Disagree | Neither agree nor disagree | Agree | Strongly agree |
|  |  |  |  |  |
| 1. I am clear about how important the disadvantages are to me in the decision about colon cancer screening. | | | | |
|  |  |  |  |  |
| Strongly disagree | Disagree | Neither agree nor disagree | Agree | Strongly agree |
|  |  |  |  |  |
| 1. For the main options I am considering I am clear about which is more important to me (the advantages or the disadvantages) in the decision about colon cancer screening. | | | | |
|  |  |  |  |  |
| Strongly disagree | Disagree | Neither agree nor disagree | Agree | Strongly agree |
|  |  |  |  |  |
| 1. I am making this choice about colon cancer screening without any pressure from others | | | | |
|  |  |  |  |  |
| Strongly disagree | Disagree | Neither agree nor disagree | Agree | Strongly agree |
|  |  |  |  |  |
| 1. I have the right amount of support from others in making this choice about colon cancer screening | | | | |
|  |  |  |  |  |
| Strongly disagree | Disagree | Neither agree nor disagree | Agree | Strongly agree |
|  |  |  |  |  |
| 1. I have enough advice about these options for colon cancer screening. | | | | |
|  |  |  |  |  |
| Strongly disagree | Disagree | Neither agree nor disagree | Agree | Strongly agree |
|  |  |  |  |  |

| 1. I feel I have made an informed choice about colon cancer screening. | | | | |
| --- | --- | --- | --- | --- |
|  |  |  |  |  |
| Strongly disagree | Disagree | Neither agree nor disagree | Agree | Strongly agree |
|  |  |  |  |  |
| 1. My decision about colon cancer screening shows what is important to me. | | | | |
|  |  |  |  |  |
| Strongly disagree | Disagree | Neither agree nor disagree | Agree | Strongly agree |
|  |  |  |  |  |
| 1. I expect to stick with my decision about colon cancer screening. | | | | |
|  |  |  |  |  |
| Strongly disagree | Disagree | Neither agree nor disagree | Agree | Strongly agree |
|  |  |  |  |  |
| 1. I am satisfied my decision about colon cancer screening. | | | | |
|  |  |  |  |  |
| Strongly disagree | Disagree | Neither agree nor disagree | Agree | Strongly agree |
|  |  |  |  |  |
